# OLDER AGE IS ASSOCIATED WITH MORE COMPLEX PERCEPTIONS OF ROMANTIC RELATIONSHIP QUALITY DURING THE COVID-19 LOCKDOWN

**DOI:** 10.1093/geroni/igad104.2193

**Published:** 2023-12-21

**Authors:** Yochai Shavit, Roi Estlein, Roni Elran-Barak, Dikla Segel-Karpas

**Affiliations:** Stanford University, Stanford, California, United States; University of Haifia, Haifa, Hefa, Israel; University of Haifa, Haifa, HaZafon, Israel; University of Haifa, Haifa, Hefa, Israel

## Abstract

The COVID-19 pandemic and the measures taken to mitigate its devastating effects posed unparalleled challenges to romantic relationships. National lockdowns forcing couples to spend long hours in closed quarters were especially straining, yet evidence points to considerable variability in the effect of the pandemic on relationship quality. Drawing on socioemotional selectivity theory, we examined age differences in perceptions of romantic relationship quality as one source of insight into this variability. During the first lockdown in Israel, 280 adults aged 25-81 reported positive and negative qualities of their romantic relationships. Of these, 105 participants completed the survey again once lockdown restrictions were lifted. Contrary to our hypotheses based on socioemotional selectivity theory, we found no evidence for age differences in the effect of the lockdown on perceptions of relationship quality. In addition, the lockdown did not influence participants’ positive and negative perceptions of their romantic partners. However, we found that whereas people of all ages represent positive and negative qualities of their romantic partners as separate constructs, the negative association between the two is weaker for older adults compared to younger adults during (but not after) the lockdown. This finding suggests that in stressful times, older adults are better able to avoid negative perceptions clouding positive perceptions and see positive aspects of relationships with romantic partners in the face of negative ones. Findings extend evidence for age associations with complex emotional experiences to emotional aspects of interpersonal relationships and may inform potential interventions for improving romantic relationships during stressful times.

